# Current Approach to Successful Liberation from Renal Replacement Therapy in Critically Ill Patients with Severe Acute Kidney Injury: The Quest for Biomarkers Continues

**DOI:** 10.1007/s40291-020-00498-z

**Published:** 2020-10-24

**Authors:** Helmut Schiffl, Susanne M. Lang

**Affiliations:** 1 Department of Internal Medicine IV, University Hospital LMU Munich, Munich, Germany; 2 Klinik für Innere Medizin II, Universitätsklinikum Jena, Jena, Germany

## Abstract

Recovery of sufficient kidney function to liberate patients with severe acute kidney injury (AKI-D) from renal replacement therapy (RRT) is recognized as a vital patient-centred outcome. However, no clinical consensus guideline provides specific recommendations on when and how to stop RRT in anticipation of renal recovery from AKI-D. Currently, wide variations in clinical practice regarding liberation from RRT result in early re-start of RRT to treat uraemia after premature liberation or in the unnecessary prolonged exposure of unwell patients after late liberation. Observational studies, predominantly retrospective in nature, have attempted to assess numerous surrogate markers of kidney function or of biomarkers of kidney damage to predict successful liberation from RRT. However, a substantial heterogeneity in the timing of measurement and cut-off values of most biomarkers across studies allows no pooling of data, and impedes the comparison of outcomes from such studies. The accuracy of most traditional and novel biomarkers cannot be assessed reliably. Currently, the decision to discontinue RRT in AKI-D patients relies on daily clinical assessments of the patient’s status supplemented by measurement of creatinine clearance (> 15 ml/min) and 24-h urine output (> 2000 ml/min with diuretics). Clinical trials objectively comparing the success of validated biomarkers for guiding optimal timed liberation from RRT in AKI-D will be required to provide high-quality evidence for guidelines.

## Key Points

**Table:** 

Successful liberation from renal replacement therapy in critically ill patients is a complex individualized approach.
Novel kidney biomarkers appear to have a promising discriminatory capability and may aid the assessment of renal recovery from AKI and safe liberation from renal replacement therapy, either alone or in combination with other variables.

## Background

Recovery of native kidney function after an episode of severe acute kidney injury (AKI-D) requiring acute renal replacement therapy (RTT) is recognized as a vital patient-centred outcome for survivors of AKI-D. The once-conventional wisdom that AKI is a predominantly entirely reversible condition has been challenged. Patient demographic factors (age, sex, race), pre-existing renal diseases (reduced eGFR or pre-admission proteinuria) and other co-morbidities, severity of acute disease (multiorgan failure), or the frequency, severity, type and duration of AKI, and possibly RRT (intensity, dialyser membrane) are independent risk factors for maladaptive repair and de novo chronic kidney disease or end-stage renal disease (ESRD) [1–7]. Depending on the absence of pre-existing chronic kidney disease (CKD), 70–90% of the patients surviving critical illness may experience complete recovery (defined as return to pre-insult estimated or measured glomerular filtration rate). Other patients recover sufficient kidney function to discontinue dialysis (partial recovery of kidney function, or reduction in AKI stage) within the first 3 months after commencement of RRT or discharge from the hospital. Patients discharged with dialysis dependency or partial kidney recovery may improve kidney function as late as up to 1 year after discharge [8]. The degree of renal recovery, i.e. complete, partial or non-recovery (loss of renal function, ESRD) substantially affects both long-term renal and clinical prognosis. Patients with transition to ESRD via AKI-D experience early death, a high burden of cardiovascular morbidity and a poor quality of life, along with increased healthcare costs. AKI-D patients with partial recovery of renal function have lower adjusted mortality compared to those who did not recover renal function [9], but are at higher risk for death compared to patients with complete renal recovery [10]. Cohort studies with survivors of AKI-D have established important associations with the status of post-AKI kidney function. Patients with persistent AKI or pseudo-recovery of kidney function (lower serum creatinine concentration after weight loss due to critical illness) have a higher risk of experiencing subsequent episodes of AKI, re-hospitalisation, de novo CKD or progression of pre-existing CKD, morbidity and mortality from cardiovascular diseases, cancer and ESRD [11]. Currently, measures to promote kidney function recovery from AKI-D are an unmet clinical need.

## Renal Replacement Therapy and Severe Acute Kidney Injury: Benefits and Risks

Renal replacement techniques, particularly continuous haemodiafiltration and intermittent haemodialysis (IHD), are the cornerstone of supportive management of severe AKI-D. RRT is increasingly used in ICU patients to substitute excretory kidney functions while awaiting sufficient recovery of kidney function from AKI-D. The primary goals of RRT are: (a) to correct of metabolic, electrolyte and acid–base abnormalities, (b) to optimize volume status and allow parenteral nutrition and antibiotic administration, and (c) to allow recovery from renal injury and other organ damage. RRTs are life-saving procedures. However, these techniques do not come without their own problems. They may prolong the duration of severe AKI-D or impede complete recovery of native kidney function [12]. The risk for untoward effects associated with RRT mandate the clinicians to detect the earliest time-point when recovery of native kidney function is sufficient to discontinue RRT.

The objective of this narrative review is a critical appraisal of clinical, physiological and biochemical parameters for assessing recovery of native kidney function and testing the optimal timing for liberation from RRT.

## Current Practice of Liberation from RRT in AKI-D Patients

Readiness testing is the evaluation of objective clinical and kidney function criteria to determine whether a patient may be able to be successfully and safely liberated from RRT. Liberation describes the process of immediate discontinuation of full renal support that the patient receives from RRT. Weaning refers to the attempt to gradually decrease the amount of support that the patient receives from RRT (i.e. shift from continuous modes of RRT to IHD or decreasing the frequency of IHD).

The success or failure of the approach for liberation impacts on the outcome of survivors. Patients who liberate successfully may have less morbidity, mortality and resource utilization than patients who require prolonged RRT or reinstitution of RRT [13–15].

No clinical guidelines currently provide clear recommendations on the optimal timing of liberation from acute RRT in AKI-D patients recovering renal function [16, 17]. The uncertainty regarding a safe and successful liberation strategy from RRT results in heterogeneous care, which is not driven by the best evidence but by empiricism and local logistics. Nephrologists/intensivist physicians decide in an individual case, based on bedside parameters (wait and see or go-fast approaches) or based on logistic reasons (clotted circuit, in-hospital transfer for diagnostic procedures). However, premature discontinuation of RRT may result in reoccurrence of acute life-threatening uraemia requiring re-institution of RRT. Conversely, delayed liberation from RRT carries the risk for unnecessary exposure of fragile patients to the complications of RRT [18].

The wide variation in clinical practice regarding the optimal liberation strategy is illustrated by large randomized controlled trials (RCTs) that used varied urine output (UO) cut-off criteria for the decision on RRT discontinuation. The VA/ATN trial network defined recovery of kidney function on the basis of creatinine clearance, measured with the use of 6-hourly timed urine collection when urine flow increased to more than 30 ml/h or when there was a spontaneous fall in serum creatinine. RRT was discontinued if the creatinine clearance was greater than 20 ml/min [19]. Decisions regarding discontinuation of RRT for values between 12 ml/min and 20 ml/min were left to the attending clinicians. In the Artificial Kidney Initiation in Kidney Injury (AKiKI) trial, liberation from RRT was highly recommended when the UO was higher than 1000 ml/24 h (spontaneous) or higher than 2000 ml/24 h (with diuretics), and mandatory if diuresis was sufficient to allow a spontaneous decrease in serum creatinine concentration [20]. In the Effect of Early vs Delayed Initiation of Renal Replacement Therapy in critically ill patients with acute kidney injury trial (ELAIN trial), renal recovery was defined by a UO greater than 400 ml/24 h without diuretics, by a UO greater than 2100 ml/24 h with diuretic treatment and creatinine clearances greater than 20 ml/min [21]. Regrettably, none of these large clinical trials reported success rates of liberation from RRT. The multiplicity of liberation criteria used in these landmark multicentre RCTs, as well as the discrepant thresholds make it difficult to compare outcomes from these trials.

At present, the criteria for defining “successful discontinuation” vary across published studies. Most authors defined successful liberation from RRT as a specified period during which the patient did not receive further RRT. However, these periods varied considerably and ranged from 3 to 60 days.

There are several reasonable definitions [22]. Successful renal recovery to independence from RRT at 28 days may be defined by an AKI-D patient receiving no RRT by 28 days. This time-point was chosen because it is the furthest time-point from randomization where data can be obtained from the greatest number of patients participating in the studies.

Another definition of sustained recovery to dialysis independence is that AKI-D patients receive no RRT for 7 days (the most frequently used period).

Furthermore, successful cessation of RRT may be defined by no need to have dialysis for 14 days or until either death or the end of the observation period.

## Predictive Ability of Traditional Surrogate Markers of Renal Function or Novel Kidney Biomarkers for Recovery of Sufficient Renal Function in Patients with AKI-D

Numerous parameters, including traditional surrogate markers of renal function as well as novel kidney biomarkers [23], have been reported to predict successful liberation from continuous RRT or intermittent RRT (Table 1).

**Table 1:** Traditional surrogate markers of kidney function and novel kidney biomarkers to predict recovery of kidney function from acute kidney injury

Traditional markers of kidney functional	Novel biomarkers (kidney function, tubular damage/regeneration)
Serum creatinine-based criteria:	Plasma/serum criteria:
Spontaneous decrease in serum creatinine	Cystatin C
	NGAL
Decrease in serum creatinine ratio (serum creatinine day x/day 0)	Pro BNP
	Interleukin 6
eGFR	Interleukin 18
Kinetic eGFR	Osteopontin
Urine-based criteria:	Urinary criteria:
Urine output	NGAL
Urine creatinine excretion rate	Interleukin 18
Urine urea excretion rate	
Measured GFR surrogate	
Timed creatinine clearance	

### Limitations/Deficiencies of Published Data

The translation of the results from cohort studies to daily clinical practice is hampered by considerable limitations and deficiencies in the available studies.

First, the retrospective/prospective single-centre cohort studies may suffer from of residual confounding and bias. Most studies utilized small sample sizes and heterogeneous study populations (severity of underlying illness, cause of AKI, and burden of co-morbid disorders). There were wide variations in time-points of sampling even for the same variable. The authors used various definitions of successful discontinuation of RRT. All studies lacked prospective and external validation of the threshold of the surrogate marker or kidney biomarker. Only a minority of studies reported sensitivity and specificity. Few variables [UO, creatinine clearance, neutrophil gelatinase-associated lipocalin (NGAL)] were tested in at least two studies (Tables 2, 3 and 4).

**Table 2:** Urine output and predictive ability for successful liberation from acute kidney injury

Author	Study design	Study patients	Cut off	Sensitivity	Specificity	Success/failure
Aniort [34]	Retrospective	67	8.6 ml/kg	0.89	0.73	37/30
Chen [37]	Prospective	110	695 ml/day	0.83	0.89	78/32
Kim [35]	Prospective	110	12.6 ml/h/kg	0.60	0.67	89/21
Uchino [14]	Post-Hoc	1006	436 ml/day	0.46	0.81	313/216
Jeon [28]	Retrospective	1176	131 ml/day	0.81	0.72	517/659
Wu [2]	Retrospective	52	880 ml/day	0.88	1.00	9/9
Raurich [29]	Retrospective	86	178 ml/6 h	0.90	0.80	69/17
Viallet [15]	Retrospective	54	2575 ml/day	0.38	0.99	26/28
Yoshida [30]	Retrospective	52	1759 ml/day	0.76	0.79	38/14

**Table 3:** Creatinine clearance to predict recovery of renal function upon liberation of renal replacement therapy from acute kidney injury

Author	Fröhlich [31]	Stads [13]
Study design	Retrospective	Prospective
Study patients	85	92
Cut-off	23 ml/min	11 ml/min
Sensitivity	0.76	0.84
Specificity	0.84	0.68
Success/failure	53/32	61/32

**Table 4:** Novel kidney biomarkers used to predict successful liberation from renal replacement therapy

Biomarker	Author	Study design	Study patients	Cut-off	Sensitivity	Specificity	Success/failure
Cystatin C							
Serum	Kim [35]	Prospective	110	1.86 mg/l	0.76	0.63	89/21
Serum	Yang [36]	Prospective	69	2.47 mg/dl	0.95	0.540	50/19
NGAL							
Serum	Chen [37]	Prospective	110	403 ng/ml	0.91	0.61	78/32
Urine	Thomsen [38]	Prospective	59	1,650 µg/l	0.86	0.73	22/32

*NGAL* neutrophil gelatinase-associated lipocalin

Second, RRT liberation was mostly not based on standardized assessment criteria. The decision was made by the attending physician based on empiricism.

Third, both traditional markers of renal function, such as serum creatinine concentration (muscle mass, functional activity, tubular secretion, volume status) and UO (diuretics, medications, systemic inflammation, tubular dysfunction and fluid status), as well as novel kidney biomarkers are significantly affected by extrarenal factors. NGAL is one of the critical components of bacterial defence mechanisms in the circulation. NGAL concentration in the circulation markedly increases in response to bacterial infection. NGAL is mostly filtered by the glomerulus and then reabsorbed by proximal tubules. Markedly elevated serum NGAL concentrations may overload the absorption capacity of tubular epithelia, and excess NGAL proteins emerge in the urine. Thus, the utilization of NGAL in predicting onset of AKI or renal recovery from AKI in sepsis patients has been questioned. Other inflammatory disorders, like cancer or atherosclerosis, also limit the accuracy of NGAL as a marker of AKI [24]. The rise or fall of serum cystatin C may occur before the corresponding changes in serum creatinine become apparent. However, malignancy, corticosteroids and thyroid dysfunction may lead to cystatin C variability [25]. Urine IL-18 holds promise as a biomarker for the early detection of AKI. IL-18 can be induced in the proximal tubule after AKI and released into the urine. On the other hand, IL-18 can also be derived from lung injury and myocardial ischemia. Urinary IL-18 levels are markedly elevated not only in patients with acute tubular necrosis but also in a variety of other renal pathologies, including urinary tract infection or chronic renal insufficiency [26].

Fourth, the assessment of renal recovery for AKI- D patients may differ between continuous and intermittent RRT. Small solutes such as serum creatinine and blood urea nitrogen concentrations of patients treated with intermittent RRT are cleared from the blood and are in a non-steady state, complicating the use of timed excretion rates or clearances. Therefore, 24-h excretion rates used to assess recovering native kidney function need to be done during the interdialytic period on non-dialysis days and by measurement of serum creatinine at two time-points. By contrast, steady-state concentrations of this surrogate marker during continuous renal replacement therapy (CRRT) allow calculations of creatinine clearance at 2, 6 or 24 h. Generally, creatinine clearance values obtained from patients with AKI overestimate the true glomerular filtration rate due to tubular creatinine secretion. To achieve a more accurate creatinine clearance the kinetic estimated GFR has been used in small study populations [16, 18].

### Utility of Surrogate Markers of Renal Function or Novel Kidney Biomarkers for the Optimal Timing of Liberation from RRT

Katulka et al. conducted a systematic review and meta-analysis to identify predictors of successful liberation from acute RRT in critically ill patients with AKI D [16]. Twenty-three cohort studies (18 full-text articles, five conference abstracts and two case–control studies) fulfilled the inclusion criteria. All studies were observational in nature: None of the 16 biomarkers evaluated in various studies was externally validated. UO prior to discontinuation of RRT was the most described predictor (nine studies). The authors found a pooled sensitivity of 66% and specificity of 74%. Estimation of an optimal threshold to discriminate successful liberation from RRT was not feasible due to variation across studies with thresholds ranging from 191 to 1738 ml/24 h. This meta-analysis reaffirmed the importance of UO as a traditional (clinical) marker to help guide RRT liberation, but there is little evidence to determine optimal cut-off values that can be reliably used by the attending clinicians.

Moreover, the traditional criterion UO (400–500 ml/24 h without diuretics) does not seem to be sufficient on its own to guide the optimal timing of liberation from RRT in patients with AKI-D. Utilizing an standardized clinical assessment and management plan that functioned as a decision-making algorithm, Mendu et al. conducted a prospective cohort study to assess the adherence of front-line clinicians caring for ICU patients with AKI D [27]. Discontinuation of RRT was recommended based on prespecified UO thresholds of > 500 ml/24 h. Despite these recommendations, clinicians chose to discontinue RRT in only 33% of such cases. The most common reasons for not stopping RRT were volume overload, worsening serum creatinine and uraemia.

The association between diuretic-induced UO and successful discontinuation of RRT has been evaluated rarely. The findings of these studies were variable. Certainly, diuretics may make liberation from RRT easier in volume-overloaded AKI patients. Uchino et al. reported that for patients on diuretics, a UO threshold of 2330 ml/ 24 h had the highest sensitivity and specificity. This threshold compared to a cut-off value of 400 ml/24 h in patients not receiving diuretics showed a higher positive predictive value (88% vs. 81%) [14].

Jeon et al. found higher sensitivity (81% vs. 72%), specificity (72% vs. 69%) and AUC-ROC values (0.82 vs. 0.75) for non-oliguric patients receiving diuretics (*n* = 557) compared to oliguric patients treated with diuretics (*n* = 619) [28]. Raurich et al. reported that the optimal cut-off to predict weaning for 6-h UO after CRRT discontinuation was 178 ml with a sensitivity of 90%, a specificity of 91%, a positive predictive value of 90% and a negative predictive value of 90%. The AUC -ROC for 6-h UO after the weaning test to predict successful liberation were 0.94 in patients who received furosemide and 0.85 for those who did not [29]. Another study described a decrease in the predictive ability of UO following diuretic administration [30].

The collaborators of the ATN trial utilized the variable creatinine clearance for prediction of renal recovery. Patients with a 6-h creatinine clearance > 20 ml/min were trailed off RRT, whereas patients with a 6-h creatinine clearance < 12 ml/min had RRT continued [19]. Retrospective single-centre studies indicated that a creatinine clearance of 20 ml/min could accurately predict successful liberation from continuous RRT [31] or IHD [32]. Wheeler and Tolwani retrospectively evaluated the utility of the 24-h creatinine clearance cut-off value of 15 ml/min obtained while on CRRT for RRT liberation from AKI-D [33]. Eight of the nine patients (89%) in the creatinine-clearance > 15 ml/ min group successfully remained off RRT for at least 2 weeks following CRRT cessation whereas only four of the 14 patients in the creatinine clearance < 15 ml/min group successfully remained off RRT. Curve characteristics demonstrated that a threshold > 15 ml/min predicted successful liberation from CRRT. Viallet et al. analyzed traditional biomarkers (24-h urinary creatinine and urea excretion, creatinine and urea generation rate, 24-h diuresis), clinical parameters of critical illness (Simplified Acute Physiology Score, Sequential Organ Failure score) to assess which of these variables were the most significant ones for the prediction of successful RRT liberation in patients with AKI-D [15]. By multivariate analysis, a 24-h creatinine clearance of 15 ml /min was the most powerful parameter for RRT discontinuation. It had a negative predictive value of 82% and a positive predictive value of 84%. The prospective multicentre observation study by Stads et al. aimed to identify renal and non-renal predictors of successful short-term discontinuation in 92 ICU patients in whom CRRT was stopped because renal recovery was expected [13]. Discontinuation was successful in 61 of 92 patients. Patients with successful liberation of CRRT had higher day 2 UO, better renal function indicated by higher 6-h creatinine clearances or lower creatinine ratio day 2/day 0, lower urinary NGAL, shorter duration of CRRT and lower cumulative fluid balance. In the multivariate analysis, native kidney function determined by creatinine clearance or by creatinine ratio and non-renal sequential organ failure assessment were independently associated with successful discontinuation of CRRT. The area under the curve of creatinine clearance to predict successful liberation was 0.79 and the optimal cut-off value was 11 ml/min (Table 3) [13].

Kinetic GFR has been used in small populations of selected patients to achieve a more accurate contemporaneous creatinine clearance for critically ill patients. However, kinetic GFR has not gained widespread use yet. In the study by Yoshida et al. the multivariate model used to predict successful liberation from RRT included UO at day 0 (the day of CRRT cessation) and kinetic GFR at day 1 [30]. The area under the receiver-operating characteristic curve (AUROC) was 0.95 for the prediction of successful RRT discontinuation.

Individual studies reported insufficient or conflicting data on 24-h urinary creatinine or urea excretion rate. Currently, no meaningful assessment of these variables is possible [15, 34].

### Novel Kidney and Non-Renal Biomarkers for Assessment of Recovery of Kidney Function

Novel biomarkers for kidney function (serum cystatin C, proBNP, osteopontin) or kidney damage (NGAL, interleukins 6 and 18) have shown promise for prognostication of AKI and have been assessed in studies evaluating RRT discontinuation as well as kidney function recovery and kidney damage repair (Tables 1 and 4).

Serum cystatin C was the most studied novel biomarker, and this variable appeared promising for guiding discontinuation [35]. The small prospective study by Yang et al. aimed to explore the prognostic value of serum osteopontin, serum interleukin 6, serum cystatin C, serum interleukin 18 and urinary interleukin 18 and NGAL. Upon discontinuation of RRT, serum cystatin C showed the greatest predictive ability for long-term renal recovery [36].

Chen et al. conducted a prospective observational study to determine the optimal timing for discontinuation of CRRT by evaluating serum NGAL in critically ill patients with AKI-D [37]. The total of 110 patients were divided into success and failure groups according to their requirement for renewed RRT within 7 days after discontinuation. Serum NGAL was a significant predictor for successful liberation from CRRT in non-septic patients. However, UO rather than serum NGAL was a significant predictor for the optimal time of CRRT discontinuation in septic patients.

A small prospective study investigated whether urine NGAL and UO alone or in combination could be used as predictors of successful discontinuation of CRRT in 54 critically ill patients. Twenty-two of the 54 patients recovered renal function defined as dialysis independency at 72 h, while 32 did not. With a predictive value of 93% the combination of urine NGAL at 6 h after CRRT cessation and the 24-h UO prior to CRRT cessation proved to be the best diagnostic test for CRRT liberation [38].

In a small retrospective cohort study, 69 patients were analyzed to determine the predictive ability of plasma NGAL and plasma B-type natriuretic peptide (pro BNP) for subsequent recovery of kidney function. Recovery was defined as alive and independent from dialysis at 60 days [39]. Brain natriuretic peptide, a neuropeptide hormone released from myocytes in response to ventricular stretching, is a well-known biomarker of cardiac volume load, but it has been recently evaluated as an AKI biomarker for earlier assessment of intravascular volume and renal function. Twenty-nine patients (42%) recovered from AKI. Neither plasma NGAL nor pro BNP alone or in combination were accurate predictors of renal recovery. However, plasma NGAL and pro BNP improved the accuracy of clinical parameters in predicting AKI recovery. Kim et al. conducted a prospective cohort study of 110 patients who had received CRRT and were weaned from CRRT after renal recovery. Patients were considered to have been successfully weaned from CRRT once there was no need for RRT [35]. Eighty-nine patients (81%) were successfully weaned from CRRT. These patients had lower serum cysteine C levels and higher UO than the group that restarted RRT at the time of cessation of CRRT. However, the levels of serum creatinine and plasma NGAL were not significantly lower in the successful group compared to the restart group. The prospective observational study by Stads et al. found that urinary NGAL was lower in patients with successful discontinuation of RRT (both CRRT and IHD) [40]. These data suggested less kidney damage. However, the association between urinary NGAL and successful discontinuation was not significant in the multivariate analysis.

The prospective study with 102 ICU patients with AKI-D by Yang et al. aimed to explore the prognostic value of several biomarkers (serum osteopontin, interleukin 6, interleukin 18, cystatin C and serum NGAL; urinary NGAL and interleukin 18) measured upon discontinuation of RRT for their value in predicting 60-day survival and renal recovery [36]. The authors concluded that the serum levels osteopontin, interleukin 6 and cystatin C may be useful combined with conventional predictors when considering withdrawal from RRT.

## Update of Biomarker-Guided Timing of RRT Liberation

The use of novel biomarkers remains largely experimental at present, and so far cannot be used as a primary predictor for liberation from RRT. While there are no validated novel biomarkers for prediction of the optimal timing of RRT liberation, preliminary evidence suggests that both functional and damaged biomarkers can aid the assessment of recovery of kidney function and help to guide the process of RRT liberation.

## Criteria for RRT Liberation in Anticipation of Renal Recovery in AKI-D

Previous narrative reviews and a recent editorial emphasized the importance of daily clinical assessment while considering RRT liberation for AKI-D patients [17, 18, 41]. A timed creatinine clearance > 15 ml/min or a UO > 2000 ml/min with diuretics, and prevention of a second renal hit and hypervolemia during the early post-liberation period may contribute to successful liberation (Table 5).

**Table 5:** Criteria for optimal timing of liberation from renal replacement therapy in anticipation of kidney recovery (RRT)

Readiness for de-escalation
Resolution of precipitating acute events
Reduction in acuity and improvement in multiorgan dysfunction
Capability to maintain metabolic, electrolyte, acid–base homeostasis by conservative therapy
Increasing urine output (> 400 ml/24 h)
Biomarker-guided attempt to discontinue RRT
Timed (6-h or 24-h) creatinine clearance > 15 ml/min
Diuretic-induced urine output > 2000 ml/min
Outcomes
Days off RRT
Short-term morbidity and mortality
Need for re-initiation of RRT (failure)
Assessment of the attempt
Success or failure

## Implications for Future Research

To date, a wide range of different candidate markers have been evaluated predominantly in small, single-centre, retrospective studies encompassing mixed populations of critically ill patients with severe AKI requiring continuous RRT. There is a need for replication of the most promising surrogate markers (timed UO, creatinine clearance) or novel kidney biomarkers (serum cystatin C and NGAL) in larger prospective randomized trials of selected ICU patients with a high pre-test probability of renal recovery (normal pre-AKI eGFR, no pre-existing proteinuria, low burden of co-morbid disorders). The diagnostic utility of a biomarker should be reported not only by specificity and sensitivity but also by its positive predictive value and negative predictive value. The candidate biomarker should predict renal recovery with high precision and should not be affected by confounding variables (infection, medication usage).

Further work is needed to reduce the heterogeneity of reported cut-offs by ideal measurements of the intervals (repeated or serial determinations) and selection of patients. The overall feasibility of the candidate biomarkers should be validated in the real-word setting. At present, classical surrogate markers or novel biomarkers of kidney function seem to be more promising than biomarkers of kidney damage.

## Conclusions

Given the limitations of the sparse literature on this topic, it is evident that there are no consensus criteria regarding successful RRT discontinuation. The establishment of generally accepted liberation criteria is a necessary step for RRT discontinuation decision making. Evidence-based and successful liberation from RRT could potentially improve patient outcomes by limiting RRT duration.

## Declarations

### Data availability statement

Data sharing is not applicable to this article as no new datasets were generated or analyzed.
