# Correction to: Current Approach to Successful Liberation from Renal Replacement Therapy in Critically Ill Patients with Severe Acute Kidney Injury: The Quest for Biomarkers Continues

**DOI:** 10.1007/s40291-021-00536-4

**Published:** 2021-06-03

**Authors:** Helmut Schiffl, Susanne M. Lang

**Affiliations:** 1 Department of Internal Medicine IV, University Hospital LMU Munich, Munich, Germany; 2 Klinik für Innere Medizin II, Universitätsklinikum Jena, Jena, Germany

## Correction to: Molecular Diagnosis & Therapy (2021) 25:1–8 https://doi.org/10.1007/s40291-020-00498-z

The article Current Approach to Successful Liberation from Renal Replacement Therapy in Critically Ill Patients with Severe Acute Kidney Injury: The Quest for Biomarkers Continues written by Helmut Schiffl, Susanne M. Lang was originally published Online First without Open Access. After publication in volume 25, issue 1 the author decided to opt for Open Choice and to make the article an Open Access publication. Therefore, the copyright of the article has been changed to ©The Author(s) and the article is forthwith distributed under the terms of the Creative Commons Attribution.

**Funding** Open Access funding provided by Projekt DEAL.

